# Early transcriptomic response of innate immune cells to subcutaneous BCG vaccination of mice

**DOI:** 10.1186/s13104-024-06901-w

**Published:** 2024-09-09

**Authors:** Liya Kondratyeva, Alexey Kuzmich, Irina Linge, Victor Pleshkan, Olga Rakitina, Sofia Kondratieva, Eugene Snezhkov, Alexander Sass, Irina Alekseenko

**Affiliations:** 1 Shemyakin-Ovchinnikov Institute of Bioorganic Chemistry of the Russian Academy of Sciences, Moscow, 117997 Russia; 2 Central Tuberculosis Research Institute, Moscow, 107564 Russia

**Keywords:** RNA-seq, Transcriptomes, Immune cells, Innate immunity, Trained immunity, BCG, Vaccination, C57BL

## Abstract

**Objectives:**

Current data suggests that Bacille Calmette-Guerin (BCG) vaccination contributes to nonspecific enhancement of resistance to various infections. Thus, BCG vaccination induces both specific immunity against mycobacteria and non-specific “trained immunity” against various pathogens. To understand the fundamental mechanisms of “trained” immunity, studies of transcriptome changes occurring during BCG vaccination in innate immunity cells, as well as in their precursors, are necessary. Furthermore, this data possesses important significance for practical applications associated with the development of recombinant BCG strains aimed to enhance innate immunity against diverse infectious agents.

**Data description:**

We performed RNA sequencing of innate immune cells derived from murine bone marrow and spleen three days after subcutaneous BCG vaccination. Using fluorescence-activated cell sorting we obtained three cell populations for each mouse from both control and BCG vaccinated groups: bone marrow monocytes and neutrophils and splenic NK-cells. Then double-indexed cDNA libraries for Illumina sequencing from the collected samples were prepared, the resulting cDNA library mix was subjected to NovaSeq 6000 sequencing. This paper describes the collection of 24 RNA sequencing samples comprising 4 sets of immune cell populations obtained from subcutaneously BCG-vaccinated and control mice

## Objective

The increasing amount of data from epidemiological and immunological studies indicates that, in addition to the target specific effects against particular diseases, vaccines might exhibit off-target heterologous effects on other unrelated pathogens [1, 2]. Research and randomized clinical trials (reviewed in [3]) demonstrate that the use of Bacillus Calmette-Guérin (BCG) contributes to increased resistance not only to tuberculosis but also to other diseases, resulting in a reduction in mortality from these conditions [3–7]. This effect is characteristic of other vaccines as well, and it is primarily associated with the training of innate immune cells (to form “trained immunity” [8]), that occurs during the primary infection or vaccination [9–11]. Subsequently, these trained cells provide protection against secondary infection through mechanisms independent of adaptive T and B cell responses [9]. Moreover, evidence suggest that “trained” innate immune cells may exhibit enhanced antiviral and anticancer activity [5, 12, 13]. In vitro and in vivo experiments demonstrated that the exposure of different types of cells to BCG led to the acquirement of characteristic features of “trained” cells by monocytes, macrophages, natural killers, neutrophils, and eosinophils [14]. It was also demonstrated that BCG vaccination induces epigenetic reprogramming of hematopoietic stem cells in the bone marrow, leading to subsequent transcriptional and functional changes, resulting in the emergence of “trained” myeloid cells such as monocytes and neutrophils [10, 15]. These observations explain the mechanism behind the prolonged presence of “trained” immune cells in the bloodstream after vaccination. In order to understand the fundamental mechanisms of “trained” immunity, studies of transcriptome changes occurring during BCG vaccination in innate immunity cells, as well as in their precursors, are necessary. Furthermore, this data possesses important significance for practical applications associated with the development of recombinant BCG strains aimed to enhance innate immunity against diverse infectious agents (viruses, bacteria, fungi).

### Data description

C57Bl/6J mice were subcutaneously vaccinated with 106 CFU of Mycobacterium bovis/BCG, mice in the control group were subcutaneously injected with PBS. Three days after BCG administration, vaccinated and control animals were sacrificed under isoflurane anesthesia, by exsanguination and subsequent cervical dislocation, after which spleen and bone marrow were collected. Obtained cell suspensions from spleen and bone marrow were stained with antibody panel, containing conjugated antibodies against CD45-PerCP/Cy5.5, CD11b-FITC, NK1.1-PE, Ly6G-APC, and Ly6C-APC/Cyanine. Next, cell population analysis and fluorescence-activated cell sorting (FACS) were performed. Three cellular populations, Monocytes CD45 + CD11b + Ly6C+, Neutrophils CD45 + CD11b + Ly6G+, NK-cells CD45 + CD11b-NK1.1 + for each mouse from both control and BCG vaccinated groups were collected by FACS. As a result, 300,000 neutrophils, 50,000 monocytes and 50,000 NK cells for each mouse from both groups were isolated. Moreover, 1 million of total unsorted bone marrow cells from each mouse were collected to reveal common changes for all immune cells. All obtained cell populations were further used for total RNA extraction. The RNA quality was assessed via agarose gel electrophoresis using Agilent TapeStation 4200 System. The total RNA was further depleted from rRNA and used for directed double-indexed RNA-seq library preparation. Finally, the resulting 24 individually indexed RNA-seq libraries (3 × Bone Marrow-Control, 3 × Bone Marrow-BCG, 3 × Neutrophils-Control, 3 × Neutrophils-BCG, 3 × Monocytes-Control, 3 × Monocytes-BCG, 3 × NK-Control, 3 × NK-BCG) were mixed in equimolar amounts, the final mixture was analyzed with Agilent TapeStation system. The median fragment length of the pooled fragments was 357 b.p., distributed between 200 and 700 b.p. (Table 1)

The resulting RNA-seq library mixture was further subjected to NovaSeq 6000 (Illumina) sequencing. Overall, 1,418,200,000 double-end 150 b.p. reads were obtained. The fastq files were analyzed with FASTQC tool [16], the resulting report indicated acceptable sequencing quality for all 24 samples. The mean amount of paired-end reads per library was 57 million. The reads were further aligned to murine reference genome (GRCm38/mm10) via HISAT2 [16] tool. The successfully aligned reads were then counted with featureCount tool [17] (GENCODE vM23, GRCm38.p6 was used as the reference transcriptome, FeatureCount table accessible onhttps://identifiers.org/geo:GSE261448 [18]).

**Table 1:** Overview of data files/data sets

Label	Name of data file/data set	File types (file extension)	Data repository and identifier (DOI or accession number)
Data files 1	Raw sequence reads for NK cell sample from control mice	FASTQ files (.fastq.gz)	NCBI GEO; https://identifiers.org/geo:GSM8144250 [19]
Data files 2	Raw sequence reads for NK cell sample from control mice	FASTQ files (.fastq.gz)	NCBI GEO; https://identifiers.org/geo:GSM8144252 [20]
Data files 3	Raw sequence reads for NK cell sample from control mice	FASTQ files (.fastq.gz)	NCBI GEO; https://identifiers.org/geo:GSM8144254 [21]
Data files 4	Raw sequence reads for NK cell sample from BCG-vaccinated mice	FASTQ files (.fastq.gz)	NCBI GEO; https://identifiers.org/geo:GSM8144251 [22]
Data files 5	Raw sequence reads for NK cell sample from BCG-vaccinated mice	FASTQ files (.fastq.gz)	NCBI GEO; https://identifiers.org/geo:GSM8144253 [23]
Data files 6	Raw sequence reads for NK cell sample from BCG-vaccinated mice	FASTQ files (.fastq.gz)	NCBI GEO; https://identifiers.org/geo:GSM8144255 [24]
Data files 7	Raw sequence reads for Neutrophils sample from control mice	FASTQ files (.fastq.gz)	NCBI GEO; https://identifiers.org/geo:GSM8144256 [25]
Data files 8	Raw sequence reads for Neutrophils sample from control mice	FASTQ files (.fastq.gz)	NCBI GEO; https://identifiers.org/geo:GSM8144258 [26]
Data files 9	Raw sequence reads for Neutrophils sample from control mice	FASTQ files (.fastq.gz)	NCBI GEO; https://identifiers.org/geo:GSM8144260 [27]
Data files 10	Raw sequence reads for Neutrophils sample from BCG-vaccinated mice	FASTQ files (.fastq.gz)	NCBI GEO; https://identifiers.org/geo:GSM8144257 [28]
Data files 11	Raw sequence reads for Neutrophils sample from BCG-vaccinated mice	FASTQ files (.fastq.gz)	NCBI GEO; https://identifiers.org/geo:GSM8144259 [29]
Data files 12	Raw sequence reads for Neutrophils sample from BCG-vaccinated mice	FASTQ files (.fastq.gz)	NCBI GEO; https://identifiers.org/geo:GSM8144261 [30]
Data files 13	Raw sequence reads for Monocytes sample from control mice	FASTQ files (.fastq.gz)	NCBI GEO; https://identifiers.org/geo:GSM8144262 [31]
Data files 14	Raw sequence reads for Monocytes sample from control mice	FASTQ files (.fastq.gz)	NCBI GEO; https://identifiers.org/geo:GSM8144264 [32]
Data files 15	Raw sequence reads for Monocytes sample from control mice	FASTQ files (.fastq.gz)	NCBI GEO; https://identifiers.org/geo:GSM8144266 [33]
Data files 16	Raw sequence reads for Monocytes sample from BCG-vaccinated mice	FASTQ files (.fastq.gz)	NCBI GEO; https://identifiers.org/geo:GSM8144263 [34]
Data files 17	Raw sequence reads for Monocytes sample from BCG-vaccinated mice	FASTQ files (.fastq.gz)	NCBI GEO; https://identifiers.org/geo:GSM8144265 [35]
Data files 18	Raw sequence reads for Monocytes sample from BCG-vaccinated mice	FASTQ files (.fastq.gz)	NCBI GEO; https://identifiers.org/geo:GSM8144267 [36]
Data files 19	Raw sequence reads for Bone marrow sample from control mice	FASTQ files (.fastq.gz)	NCBI GEO; https://identifiers.org/geo:GSM8144268 [37]
Data files 20	Raw sequence reads for Bone marrow sample from control mice	FASTQ files (.fastq.gz)	NCBI GEO; https://identifiers.org/geo:GSM8144270 [38]
Data files 21	Raw sequence reads for Bone marrow sample from control mice	FASTQ files (.fastq.gz)	NCBI GEO; https://identifiers.org/geo:GSM8144272 [39]
Data files 22	Raw sequence reads for Bone marrow sample BCG-vaccinated mice	FASTQ files (.fastq.gz)	NCBI GEO; https://identifiers.org/geo:GSM8144269 [40]
Data files 23	Raw sequence reads for Bone marrow sample BCG-vaccinated mice	FASTQ files (.fastq.gz)	NCBI GEO; https://identifiers.org/geo:GSM8144271 [41]
Data files 24	Raw sequence reads for Bone marrow sample BCG-vaccinated mice	FASTQ files (.fastq.gz)	NCBI GEO; https://identifiers.org/geo:GSM8144273 [42]
Data file 25	FeatureCount table	Table (.txt.gz)	NCBI GEO; https://identifiers.org/geo:GSE261448 [18]

### Limitations

This study is limited by the small sample size, and technical issues resulting in batch effects further reducing the statistical power. The experimental design takes every care to obtain the maximum possible amount of information using the minimum number of animals to be conducted in accordance with national and international standards regulating the use of experimental animals for scientific purposes.

## Data Availability

The data described in this Data note can be freely and are accessible through GEO Series accession number GSE261448 (https://identifiers.org/geo:GSE261448).

## Abbreviations

BCG: Bacille Calmette-Guerin
GEO: Gene Expression Omnibus
RNA-seq: RNA-sequencing
FACS: Fluorescence-Activated Cell Sorting
CFU: Colony-Forming Unit
NK: Natural Killers
